# Hypnosis for Postoperative Pain Management After Colectomies: A Single-Centre Randomized Controlled Feasibility Trial

**DOI:** 10.1097/DCR.0000000000004384

**Published:** 2026-08-27

**Authors:** Ana-Maria Toth, Paul Mackey, Stefano Di Donato, Begonya Alcacer-Pitarch

**Affiliations:** 1 School of Health Sciences, University of Exeter, Exeter, United Kingdom; 2 Somerset NHS Foundation Trust, Taunton, United Kingdom; 3 Leeds Teaching Hospitals NHS Trust, Leeds, United Kingdom

## Abstract

**Background:**

Postoperative pain management after colectomy remains challenging, with opioid-related complications impacting recovery. This study evaluates the feasibility of implementing clinical hypnosis as an adjunctive intervention.

**Objective:**

To determine the feasibility of conducting a full-scale randomized controlled trial investigating hypnosis for post-colectomy pain management.

**Design:**

Single-centre randomized controlled feasibility trial.

**Setting:**

District general hospital in South-West England (July 2023–February 2024).

**Patients:**

Forty-one adults undergoing elective colectomy.

**Interventions:**

Participants were randomized to standard care or standard care plus three perioperative hypnosis sessions (preoperatively, first and third day post-surgery).

**Main Outcome Measures:**

Primary: recruitment rates, retention, and intervention adherence. Secondary: pain scores (Numeric Pain Scale), analgesic requirements, and complications.

**Results:**

Recruitment achieved 58% (41/71 eligible patients), with 90% retention and 92.5% intervention adherence. The hypnosis group demonstrated pain reductions approximating the minimum clinically important difference, providing signals of potential clinical benefit following sessions: at 24 hours post-surgery, median pain decreased from 5.0 to 2.5 (median difference -2.0, 95% confidence interval -2.5 to -1.0), and at Day 3, from 4.0 to 1.5 (median difference -2.5, 95% confidence interval -3.0 to -1.5). Between-group comparisons showed lower pain in the hypnosis group at 24 hours (median 5.0 vs 6.0, median difference -1.2, 95% confidence interval -2.3 to -0.1).

**Limitations:**

Single-centre design limits generalizability; participant and staff blinding was unfeasible; hypnotisability was not assessed.

**Conclusions:**

Hypnosis is feasible in acute surgical settings, with trends supporting pain reduction and positive patient experiences. A definitive trial is warranted to determine efficacy for post-colectomy pain management.

## Introduction

Colectomies involve significant postoperative pain that can impede recovery, delay mobilization, and prolong hospitalization. Additionally, suboptimal pain control increases morbidity risk and healthcare costs.^1–6^

Current Enhanced Recovery After Surgery (ERAS) guidelines emphasize multimodal analgesia incorporating pharmacologic and non-pharmacologic modalities to reduce opioid exposure and improve recovery outcomes. Non-pharmacological treatments that can modify pain and reduce opioid use are therefore in demand as part of a multimodal opioid-sparing regimen. ^6–9^

Clinical hypnosis has emerged as a promising adjunctive approach to pain management offering advantages as an inexpensive intervention with minimal adverse effects. Multiple studies and meta-analysis have documented benefits of hypnotic interventions for various surgical procedures, including reduced postoperative pain intensity and decreased analgesic requirements, and several professional societies now acknowledge its potential role in perioperative care.^3,5,7,8,10–14^

Although perioperative hypnosis has been evaluated in major abdominal surgery, procedure-specific data in colectomy patients within contemporary ERAS pathways remain limited.^3,15^ Important uncertainties remain regarding how hypnosis can be delivered and integrated around colectomy (including timing, mode of delivery, and training requirements), which pain and recovery outcomes should be prioritised, and how differences in surgical approach and complication risk should be handled when designing a definitive multicentre trial. In addition, variability in post-colectomy pain trajectories and the range of pain and recovery outcomes used in colorectal research, make it difficult to pre-specify which endpoints and timepoints should be prioritised in a definitive RCT.^16–18^

A hypnotic intervention typically consists of an induction involving mind–body relaxation and focused attention, followed by therapeutic suggestions.^19,20^

Given its multifaceted action, favourable safety profile, and compatibility with ERAS principles, hypnosis represents an attractive adjunctive therapy for complex surgical procedures such as colectomies.^21^ Colectomies were selected as the index procedure because it is a common colorectal operation associated with substantial postoperative pain, frequent opioid exposure, and well-defined ERAS pathways, making it a clinically important and methodologically suitable context in which to optimise and test perioperative hypnosis.

This feasibility RCT aimed to evaluate the practicality of conducting a full-scale RCT of perioperative clinical hypnosis for post-colectomy pain management in contemporary ERAS pathways, including recruitment, retention, intervention adherence, and implementation issues specific to colectomy and to generate preliminary signals to inform outcome selection and stratification strategies for a future definitive trial.

## Materials and Methods

### Study Design

This study was an open, pragmatic, single-centred, two-armed randomised controlled feasibility trial evaluating patient acceptability of randomisation and adherence to the hypnosis intervention in patients undergoing elective colectomy.

There were two study arms: hypnotic analgesia plus standard care (intervention arm), and standard care (control arm).

### Participants

Adults scheduled for elective colectomy at a district general hospital in South-West England (July 2023-February 2024) were recruited through surgical clinics. Inclusion: ≥18 years, English-speaking. Exclusions: emergency surgery, severe psychiatric illness, postoperative National Early Warning Score 2 NEWS ≥5, reflecting the Trust’s escalation threshold for medium-risk acute deterioration.

NEWS2 is a standardized aggregate scoring system used across the NHS to identify and respond to acutely unwell patients, with total scores of 5–6 indicating medium clinical risk and triggering urgent review.^16,22^

### Sample size

We recruited 41 patients over seven months, shorter than the initially planned 12-month period. The accelerated timeline did not appear to affect feasibility outcomes or introduce biases, hence is transparently reported. The target sample size was based on published feasibility trial recommendations of 30-70 participants to estimate variability and feasibility outcomes.^6,16^

### Randomisation

Following baseline assessments and consent, participants were randomised in a 1:1 ratio to either standard care alone (control) or standard care plus hypnosis intervention (active). Randomisation was conducted using variable block sizes via a secure, web-based REDCap module to ensure allocation concealment. Randomisation was not stratified by surgical approach (open, laparoscopic, robotic). Surgical approach was recorded and reported descriptively to inform stratification in the future definitive RCT. Due to the nature of the intervention, blinding of participants and clinical staff was not feasible, reflecting pragmatic real-world implementation constraints. Additional blinded assessors were not used due to staff resource constraints, as the study had limited charitable funding. To mitigate potential bias arising from this, care protocols were standardized across groups.

### Interventions

The hypnosis group received three structured sessions conducted by the principal investigator (trained in clinical hypnosis). Sessions were delivered using standardized scripts to ensure consistency. A structured outline of the session content (induction components, therapeutic suggestion domains, and reorientation procedures) is provided in Supplementary Appendix 1 to support reproducibility, while full verbatim scripts are not reproduced because they form part of an ongoing programme of doctoral research. Each session included an induction, therapeutic suggestions targeting pain and anxiety, and reorientation. The sessions were delivered by the principal investigator, a registered nurse who had completed accredited clinical hypnosis training and supervised practice before the trial.

We did not formally assess participants’ hypnotisability, which may affect responsiveness to hypnosis. Postoperative sessions followed the same structure. Participants received self-hypnosis training and kept diaries tracking analgesic use and pain. Additionally, all patients received the same colorectal ERAS multimodal analgesia pathway, including regular paracetamol and non-steroidal anti-inflammatory drugs, with opioids prescribed for breakthrough pain and regional techniques used according to departmental protocols in both groups. Perioperative analgesia and length of stay followed local Trust ERAS guidelines and colorectal surgery policies, with patients typically discharged between postoperative day 3 and 5 depending on recovery, and opioids prescribed according to individual clinical need and local prescribing guidance.

### Measures

#### Participants demographics and medical information

Demographic data, including gender, age in years, ethnicity, height, weight, body mass index, and information about participant’s medical history and medication was collected.

#### Primary feasibility outcomes

The feasibility of the study design and hypnosis intervention was assessed via recruitment, retention, attrition (≤20%), and adherence (≥80%) to study protocol and completeness of data collection. ^18,22^

### Secondary feasibility outcomes

#### Pain Severity

Pain scores were measured using an 11-point Numeric Pain Scale (NPS, 0-10) where 0 is a value that indicates no pain and 10 indicates the worst possible pain. Participants were asked to rate, using the Numeric Pain Score (NPS) the pain they have experienced in the last 24h and at the time of the visit. For the intervention group the pain levels before and after the hypnosis session were captured.^23^

Pain was evaluated at five timepoints: baseline, 24 hours post-operatively, 3 days post-surgery, on discharge day, and 10 days post-surgery, with two additional pain evaluations for the hypnosis group immediately after each session on days 1 and 3 to capture immediate effects.^24^

Data collection regarding medication, mobility, oral intake, and usage of the diary were recorded by PI from electronic databases, nursing notes and conversations with patients at each visit.

#### Health-related quality of life

Health-related quality of life was assessed using the EQ-5D instrument, which comprises five domains (mobility, self-care, usual activities, pain/discomfort, and anxiety/depression). Each domain is rated on a 5-level scale from “no problems” to “extreme problems.”^16,25^

#### Postoperative complications

Complications were systematically documented throughout hospitalization using the Clavien-Dindo Classification (CDC), a standardized system validated to grade postoperative complications by severity based on the intervention required to manage them. The CDC does not use predefined domains but objectively categorizes any complication (e.g., infectious, mechanical, cardiovascular, neurological, procedural) into five hierarchical grades I-V. The score ranges from I (mild) to V (fatal), with higher grades indicating greater severity and resource utilization. ^26,27^

#### Patient diaries and qualitative analysis

Participants in the hypnosis group kept a postoperative diary recording daily pain scores (NRS 0-10), analgesic use and type of pain, self-hypnosis practice and free-text comments about their recovery. Diaries were collected after the 10-day follow-up visit; however, diary completion was incomplete and only free text comments were analysed as reported below. A descriptive thematic approach was used, grouping recurring phrases into simple descriptive themes with short anonymised quotations selected to illustrate common findings in the Results section.

#### Statistical Analysis

Following an intention-to-treat approach, feasibility endpoints were summarised descriptively. Quantitative variables are presented as means (SD) or medians (IQR), as appropriate; categorical variables as counts (percentages). In line with CONSORT guidance for feasibility trials, no formal hypothesis testing was performed for feasibility outcomes. ^6^

To explore preliminary efficacy signals (secondary/exploratory outcomes), we calculated between-group mean differences with 95% confidence intervals. All between-group comparisons are exploratory in nature, with confidence intervals often crossing zero, reflecting the study’s feasibility focus rather than powered efficacy design. Where reported, *p*-values (e.g., Welch’s *t*-tests on change scores) are exploratory and interpreted cautiously, without multiplicity adjustment. Longitudinal trends in patient-reported outcomes were visualised across timepoints using means with standard errors.

Missing data was handled using an available-case strategy: all available observations contributed to descriptive summaries; change-from-baseline analyses were computed only for participants with both baseline and the relevant follow-up value. We did not perform imputation and denominators. All analyses were conducted in R (v4.3).^28^

Patient comments were grouped by recurring themes; selected quotes illustrated common views.^29,30^

#### Registrations and funding

Ethical approval for the study was given by HRA and Health and Care Research Wales (HCRW) committee on the 25/05/23/(REC reference 23/SW/0043) and all procedures followed the Helsinki Declaration principles. ClinicalTrials.gov: NCT06499909.

## Results

### Baseline Characteristics

The study groups were generally well-balanced. The hypnosis group had a slightly higher average age and a similar female-male ratio. Interestingly, the hypnosis group had substantially higher rates of comorbidities. (Table 1)

The median for the NPS scores at baseline, were the same in both groups (2 [IQR 1-3]). Surgical procedure types were similarly distributed between groups, with left-sided procedures comprising 55.0% in the control group and 50.0% in the hypnosis group. (Table 2)

### Feasibility outcomes

#### Recruitment, retention and Protocol adherence

**Figure 1:**
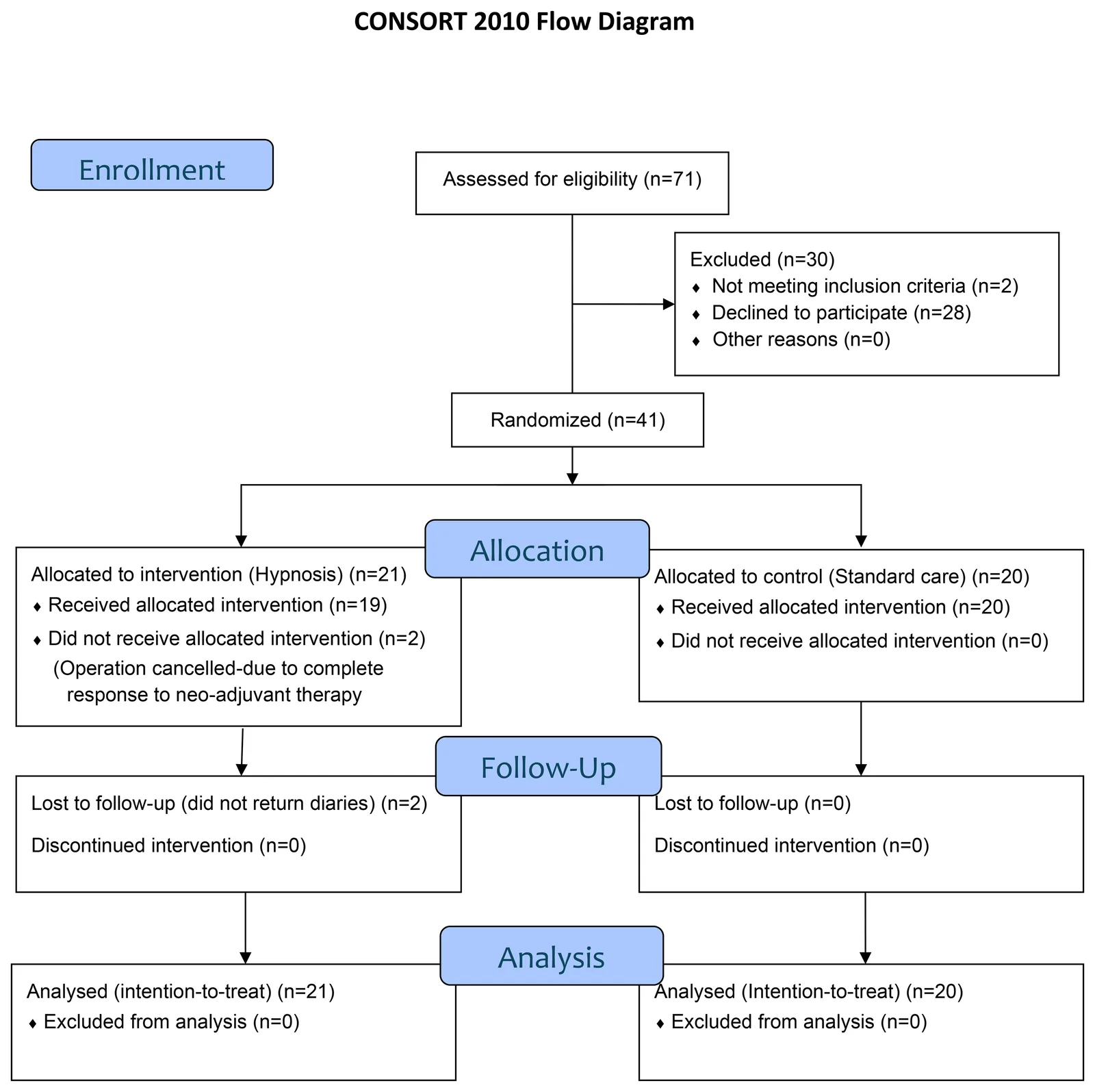
CONSORT flow diagram

Recruitment achieved 41 of 71 eligible patients (58%), exceeding the target of 40 participants with a rate of 6 participants per month, supporting feasibility of recruitment timelines.^22^ (Figure 1) Retention was 90%, with attrition (10%) due to cancelled surgeries following good therapeutic response (n=2) and loss of completed diary in post (n=2). Protocol adherence was 92.5%. Common reasons for declining were surgery-related stress and scepticism towards hypnosis, which may introduce selection bias.

### Secondary outcomes

#### Pain Scores

Pain score outcomes for both groups are summarized in Tables 3 and 4.

Between-group comparisons: the hypnosis group showed lower pain at 24 hours post-surgery (median 5.0 vs 6.0, median difference -1.2, 95% CI -2.3 to -0.1) and at discharge (median 3.5 vs 4.0, median difference -0.6, 95% CI -1.6 to 0.4) with comparable scores at other timepoints.(Table 3) Within-group effects: hypnosis produced clinically meaningful immediate pain reductions at post-operative timepoints. At 24 hours post-surgery, pain decreased from median 5.0 to 2.5 (median difference -2.0, 95% CI -2.5 to -1.0, n=17 paired observations). At Day 3, pain decreased from 4.0 to 1.5 (median difference -2.5, 95% CI -3.0 to -1.5, n=19). (Table 4)

These immediate pain reductions of 2.0-2.5 points exceed the minimum clinically important difference of 1 point on the 0-10 NRS, suggesting clinically meaningful acute analgesic effects immediately following hypnosis intervention.

#### Analgesics Use

The study groups followed similar hospital analgesia protocols, but differences in medication utilization emerged (Table 5). Both groups required higher doses of opioids on the first post-operative day, in line with usual tendencies post-surgery. However, by post-operative day 3, opioid consumption in the hypnosis group decreased substantially compared to the control group, aligning with pain reduction patterns.

#### Postoperative Complications

In the hypnosis group, 5 patients experienced postoperative complications (8 events), whereas in the control group 9 patients had complications (10 events) but the complications in the hypnosis group were generally of higher Clavien-Dindo grade (Table 6). In the hypnosis group, several events required reoperation (e.g., anastomotic leak, abdominal wall dehiscence) and unplanned HDU/ITU admission, whereas the control group predominantly experienced Clavien–Dindo grade II complications such as pneumonia, syncope requiring medication, and ileus requiring pharmacological treatment.

#### Patients’ experience about hypnosis

This analysis was intended to complement the quantitative feasibility outcomes by providing preliminary insight into acceptability and perceived usefulness of hypnosis from the patient perspective. Eleven participants (52.8%) wrote about their experience, and all reported it as positive. The most common themes were perceived reduction in pain following hypnosis, increased relaxation and stress reduction, help with surgical anxiety, and an intention to continue using hypnosis techniques beyond the study period.

#### Health-Related Quality of Life (HRQoL)

The mean change in the EQ-5D anxiety/depression domain was numerically more favourable in the hypnosis group than in controls (-0.39 vs +0.24; mean difference 0.78, 95% CI ™0.07 to 1.33; p = 0.075), suggestive of an improvement in anxiety and depression but not meeting conventional thresholds for statistical significance. No mean differences or trends were observed in the other EQ-5D domains. ^8^

#### Adverse reactions

Throughout the study, patients did not report experiencing any abreactions or side effects to the hypnosis intervention. ^31^

## Discussion

Despite advancements in multimodal analgesia, pain management after colorectal surgery remains a significant challenge, particularly due to the adverse effects associated with opioids and the variability in individual patient responses.^3^ This feasibility study supports integrating clinical hypnosis into ERAS multimodal analgesia pathways for colorectal surgery, demonstrating patient acceptability and operational feasibility. Previous perioperative hypnosis trials have shown that structured sessions can be delivered alongside routine perioperative care without major disruption to clinical workflows.^3^

### Feasibility outcomes

Recruitment achieved 41 of 71 eligible patients (58%), exceeding the target of 40 participants with a rate of 6 participants per month, consistent with or better than typical RCT recruitment rates.^32,33^ This together with 90% retention and 92.5% protocol adherence, indicates strong patient acceptability and operational feasibility of perioperative hypnosis within colorectal ERAS pathways. Enhanced pre-operative education explaining clinical hypnosis, its role in stress and anxiety reduction, and evidence from previous surgical studies may further improve recruitment in a definitive trial.^14^

### Preliminary Efficacy Signals

The clearest preliminary signal emerged from immediate pre-post hypnosis measurements, which showed acute pain reductions of approximately 2.0–2.5 points on the 0–10 NRS, exceeding the minimum clinically important difference. Between-group comparisons at 24 hours also favoured the hypnosis group and were consistent with these within-group signals, although all such analyses remain explanatory. Effects varied by timing and pain context: the pre-operative session produced no change in patients with minimal baseline pain, whereas larger reductions were seen at 24 hours and day 3 when pain was moderate to high.^15^ Beyond acute analgesia, the pre-operative session may be valuable for establishing therapeutic rapport and teaching self-hypnosis techniques, providing an additional coping tool in the postoperative period. Overall, the observed pain reductions and lower opioid consumption by day 3 in the hypnosis group should be interpreted as exploratory signals of potential acute analgesic and opioid-sparing effects that require confirmation in a definitive, adequately powered trial.

These feasibility and preliminary efficacy findings are broadly consistent with perioperative hypnosis trials and meta-analyses reporting reduced postoperative pain intensity and analgesic consumption across diverse surgical populations. The magnitude of the immediate postoperative pain reductions observed here (around 2–2.5 points on an 11-point scale) aligns with previously described clinically meaningful changes and small-to-moderate effects in prior reviews. High recruitment, retention, and adherence also mirror earlier perioperative hypnosis studies, supporting acceptability, while this trial adds colectomy-specific feasibility data within ERAS pathways.

### Complications Profile

While the hypnosis group experienced fewer overall complications (25% vs 45%), these events were of greater severity, including anastomotic leak and abdominal wall dehiscence requiring reoperation and unplanned HDU/ITU admission. In this small feasibility sample, the imbalance likely reflects chance allocation, the higher baseline comorbidity burden in the hypnosis group (85.7% vs 55.0%) and other unmeasured risk factors rather than any plausible causal effect of hypnosis. This pattern underscores the importance of stratifying by baseline complication risk and surgical complexity and of monitoring safety outcomes alongside pain and opioid use.

### Limitations

Several important limitations warrant acknowledgement. The single-centre design limits generalizability. The inability to blind participants and the use of unblinded outcome assessment by the PI introduces potential bias related to resource constraints and should be addressed in future trials by using blinded outcome assessors where possible. Delivery by a single practitioner ensured consistency but may limit generalisability; future trials should involve multiple trained practitioners with formal fidelity assessment. In addition to expectancy effects, some within-group pain reductions may partly reflect regression to the mean in patients recruited when pain was highest.

Some baseline imbalances, particularly regarding comorbidities (85.7% vs 55.0% with comorbidities), may have influenced both pain outcomes and complications, and should be considered when planning stratification and analysis in a definitive trial. Additionally, hypnotisability (individual responsiveness to hypnosis) was not measured but may moderate intervention effects; therefore future trials should assess for hypnotisability to identify better responders.

Variation in surgical approach (open, laparoscopic, robotic) may have influenced pain trajectories and complications; however, subgroup analyses were not performed as participant numbers within each surgical category were small and unevenly distributed, limiting interpretability. A definitive trial should stratify by surgical approach and pre-specify such analyses.

The small sample size limits statistical power for between-group comparisons; while within-group pre-post analysis of the hypnosis group demonstrates acute pain reductions, the lack of a powered efficacy design with hypothesis testing prevents definitive efficacy conclusions about superiority over standard care.

Missing post-hypnosis data at baseline (47.4%) and 24 hours (10.5%) resulted from competing clinical demands during perioperative periods; completion improved to 100% by Day 3. This highlights the need for protected research time in future trials. Furthermore, this pattern suggests potential bias toward underestimating immediate effects if patients with worst pain couldn’t complete post-intervention assessments. However, the progressive improvement in completion rates and 100% completion by Day 3 suggests the protocol is learnable.

Patients’ experience about hypnosis was limited to those patients that wanted/ were able to complete the diary, thus potentially introducing non-response bias. For the definitive RCT the addition of qualitative methods should be included to assess all patient experience of using hypnosis as an intervention for pain management.^30^

### Clinical Implications and Future Directions

These preliminary findings support integration of clinical hypnosis into multimodal analgesia protocols for post-colectomy patients. Differences in surgical approach are also likely to influence postoperative pain, with open colectomy generally associated with higher and more prolonged pain than minimally invasive (laparoscopic or robotic) procedures. For clinical implementation, we recommend: (1) scheduling the pre-operative session as a 1:1 personalised interaction to optimize therapeutic rapport and self-hypnosis skill acquisition; (2) delivering acute post-operative sessions when pain is moderate-to-high (particularly at 24 hours and Day 3); (3) supporting both sessions with audio recordings for between-session self-hypnosis practice; and (4) ensuring trained staff in hypnosis for the definitive RCT, achievable through accredited short-course training alongside trial-specific competency assessment.

A multicentre randomised controlled trial is warranted to definitively establish efficacy. The RCT should:

▪ Measure hypnotisability to identify responders and enable stratification or moderator analysis;
▪ Stratify by surgical approach (open vs minimally invasive) and baseline complication risk factors, and pre-specify exploratory analyses of analgesic consumption by surgical approach
▪ Employ dedicated research personnel for immediate post-intervention assessments to minimise missing data;
▪ Implement blinded outcome assessors where feasible;
▪ Include both efficacy and safety outcomes with adequate power for hypothesis testing;
▪ Extend follow-up beyond 10 days to assess sustained benefits and longer-term outcomes;
▪ Incorporate qualitative methods to explore patient experience, acceptability, and barriers to implementation;
▪ Utilize digital or remote data collection to improve data completeness

Clinical hypnosis shows promise as a non-pharmacological pain management adjunct for colorectal surgery, but efficacy remains to be definitively established through appropriately powered hypothesis-testing research.

## Conclusion

This single-centre feasibility study demonstrates that clinical hypnosis delivered perioperatively is feasible to implement in acute surgical settings. The high recruitment rate (6 participants/month), 90% retention, and 92.5% protocol adherence confirm strong patient acceptability and operational feasibility. Preliminary signals of potential benefit emerged from pain reductions at 24 hours and Day 3 post-operatively; however, given the sample size, single-centre design, and lack of blinding, these signals require confirmation through a powered RCT. Progression to a definitive multicentre RCT is therefore justified by the favourable feasibility outcomes. Findings support progression to a definitive trial with stratification by surgical approach, complication risk, hypnotisability measurement, and extended follow-up. Clinical hypnosis shows promise as an adjunct to postoperative pain management, but efficacy remains to be established through appropriately designed hypothesis-testing research.

## Figures and Tables

**Table 1:** Baseline demographics and clinical characteristics

Characteristic	Hypnosis (n=21)	Control (n=20)
Age, years (mean ± SD)	69.3 ± 9.5	66.8 ± 11.3
Sex, n (%)		
- Male	10 (47.6)	10 (50.0)
- Female	11 (52.4)	10 (50.0)
ASA classification, n (%)		
- I-II	11 (52.4)	13 (65.0)
- III-IV	10 (47.6)	7 (35.0)
Comorbidities present, n (%)	18 (85.7)	11 (55.0)
- Hypertension	12 (57.1)	8 (40.0)
- Diabetes mellitus	7 (33.3)	4 (20.0)
- Cardiac disease	5 (23.8)	3 (15.0)
- Respiratory disease	4 (19.0)	2 (10.0)
Body mass index (BMI), kg/m^2^ (mean ± SD)	28.1 ± 5.2	27.3 ± 4.6

Abbreviations: SD, standard deviation; ASA, American Society of Anaesthesiologists physical status classification.

**Table 2:** Primary Pathology and Surgical Characteristics

Characteristic	Hypnosis (n=21)	Control (n=20)
Primary pathology, n (%)		
- Colorectal cancer	14 (66.7)	13 (65.0)
- Inflammatory bowel disease	5 (23.8)	4 (20.0)
- Diverticular disease	2 (9.5)	3 (15.0)
Operation type, n (%)		
- Open colectomy	4 (19.0)	1 (5.0)
- Laparoscopic colectomy	12 (57.1)	13 (65.0)
- Laparoscopic converted to open	2 (9.5)	1 (5.0)
- Robotic-assisted colectomy	1 (4.8)	5 (25.0)
- Operation not performed due to complete clinical response to neoadjuvant therapy	2 (9.5)	0 (0.0)

**Table 3:** Pain scores at assessment timepoints

Study visits	Hypnosis Group (n=21) Median [IQR]	Control Group (n=20) Median [IQR]	Median Difference (95% CI)
Baseline (pre-surgery)	2.0 [1.0–3.0]	2.0 [1.0–3.0]	0.1 (-0.7 to 0.9)
Day 1 post-surgery	5.0 [2.5–5.5]	6.0 [4.0–7.0]	-1.2 (-2.3 to -0.1)
Day 3 post-surgery	4.0 [2.5–5.0]	4.0 [3.0–6.0]	-0.4 (-1.5 to 0.7)
At Discharge	3.5 [2.0–4.5]	4.0 [2.0–5.0]	-0.6 (-1.6 to 0.4)
10-Day Follow-up	3.0 [1.5–4.0]	2.5 [1.0–4.0]	0.3 (-0.8 to 1.4)

* For the hypnosis group, Day 1 and Day 3 values represent pre-hypnosis measurements to enable between-group comparisons at matched timepoints.

**Table 4:** Hypnosis Group Pre- and Post-Hypnosis Pain Scores

Timepoint	Pre-Hypnosis Median [IQR]	Post-Hypnosis Median [IQR]	Median Difference (95% CI)	n paired
Baseline (pre-surgery)	2.0 [1.0–3.0]	0.0 [0.0–0.0]	0.0 (-1.5 to 0.0)	10
Day 1 post-surgery	5.0 [2.5–5.5]	2.5 [2.0–3.0]	-2.0 (-2.5 to -1.0)	17
Day 3 post-surgery	4.0 [2.5–5.0]	1.5 [1.0–2.0]	-2.5 (-3.0 to -1.5)	19

* Median differences and confidence intervals are provided for descriptive purposes only and not for hypothesis testing in this feasibility study. IQR = Interquartile Range. The median difference (pre-post) values represent the median of paired differences (post-hypnosis minus pre-hypnosis for each patient), with 95% confidence intervals calculated using bootstrap resampling. n paired indicates the number of patients with complete pre- and post-hypnosis measurements.

**Table 5:** Analgesic usage in oral morphine equivalents (mg/day)

Study Visits	Hypnosis Group (n=21) Mean ± SD	Control Group (n=20) Mean ± SD	Mean Difference (95% CI) *
Baseline (pre-surgery)	54.8 ± 22.3	48.5 ± 18.7	6.3 (-6.4 to 19.0)
Day 1 post-operatively	45.2 ± 19.8	42.7 ± 16.5	2.5 (-9.5 to 14.5)
Day 3 post-operatively	32.5 ± 16.7	36.3 ± 15.2	-3.8 (-14.5 to 6.9)
Discharge day	22.7 ± 13.9	28.8 ± 14.6	-6.1 (-15.6 to 3.4)
10 days post-operatively	15.3 ± 11.2	22.5 ± 12.8	-7.2 (-15.1 to 0.7)

* Mean differences and confidence intervals are provided for descriptive purposes only and not for hypothesis testing in this feasibility study.

**Table 6:** Postoperative complications by study group using Clavien-Dindo classification

Complication	Hypnosis (n=21)	Control (n=20)
Number of complications	5 (23.8)	9 (45.0)
**Grade I**	**2 (9.5)**	**2 (10.0)**
- Superficial surgical site infection	1 (4.8)	1 (5.0)
- Self-limiting ileus	1 (4.8)	1 (5.0)
- Minor bleeding	1 (4.8)	0 (0.0)
**Grade II**	**2 (9.5)**	**6 (30.0)**
- Deep surgical site infection	1 (4.8)	1 (5.0)
- Pneumonia	0 (0.0)	1 (5.0)
- Syncope requiring medication	0 (0.0)	1 (5.0)
- Ileus requiring pharmacological treatment	1 (4.8)	3 (15.0)
**Grade IIIa (radiological drain)**	**1 (4.8)**	**1 (5.0)**
**Grade IIIb (reoperation under GA)**	**1 (4.8)**	**0 (0.0)**
**Grade IV (ICU admission)**	**2 (9.5)**	**1 (5.0)**
**Grade V (death)**	0 (0.0)	0 (0.0)
Multiple complications	1 (4.8)	0 (0.0)

Notes:

^a Minor bleeding includes PR bleeding observed in one patient from the hypnosis group.

^b Syncope events requiring pharmacological intervention were observed in one control group patient.

^c Reoperation under general anaesthesia was performed for abdominal wall dehiscence and anastomotic leak in one hypnosis group patient.

^d ICU admission was required for two patients in the hypnosis group due to severe complications, including anastomotic leak and abdominal wall dehiscence.

^e ICU admission in the control group was required for one patient due to cardiac complications.

^f Defined as ≥3 distinct complications occurring in the same patient, observed in one hypnosis group participant.

## Key Message

Perioperative hypnosis is feasible and acceptable for colectomy patients, with preliminary signals supporting pain reduction. A multicentre efficacy trial with stratification by surgical approach, hypnotisability and baseline complication risk, along with extended follow-up is warranted to establish definitive clinical benefits and support broader NHS implementation.
